# Proposed Tandem Effect of Physical Activity and Sirtuin 1 and 3 Activation in Regulating Glucose Homeostasis

**DOI:** 10.3390/ijms20194748

**Published:** 2019-09-25

**Authors:** Francesca Pacifici, Davide Di Cola, Donatella Pastore, Pasquale Abete, Fiorella Guadagni, Giulia Donadel, Alfonso Bellia, Eleonora Esposito, Chiara Salimei, Paola Sinibaldi Salimei, Camillo Ricordi, Davide Lauro, David Della-Morte

**Affiliations:** 1 Department of Systems Medicine, University of Rome “Tor Vergata”, 00133 Rome, Italy; pacifici.francesca@gmail.com (F.P.); d.pastore3@inwind.it (D.P.); donadel@uniroma2.it (G.D.); bellia@med.uniroma2.it (A.B.); d.lauro@med.uniroma2.it (D.L.); 2 Department of Biomedicine and Prevention, University of Rome Tor Vergata, 00133 Rome, Italy; davide.dicola89@gmail.com (D.D.C.); espositoeleonora@live.it (E.E.); paola.sinibaldi@uniroma2.it (P.S.S.); 3 Department of Translational Medical Sciences, University of Naples “Federico II”, 80138 Naples, Italy; p.abete@unina.it; 4 Department of Human Sciences and Quality of Life Promotion, San Raffaele Roma Open University, 00166 Rome, Italy; fiorella.guadagni@unisanraffaele.gov.it; 5 University of Rome Tor Vergata, Neuroscience, 00133 Rome, Italy; chiara.salimei@gmail.com; 6 Diabetes Research Institute (DRI) and Clinical Cell Transplant Program, University of Miami Miller School of Medicine, Miami, FL 33136, USA; cricordi@miami.edu; 7 Department of Neurology and Evelyn F. McKnight Brain Institute, Miller School of Medicine, University of Miami, Miami, FL 33136, USA

**Keywords:** sirtuins, physical activity, glucose homeostasis, diabetes, oxidative stress, inflammation

## Abstract

Sirtuins (SIRTs) are seven nicotinamide adenine dinucleotide (NAD^+^)-dependent protein deacetylases enzymes (SIRT1–7) that play an important role in maintaining cellular homeostasis. Among those, the most studied are SIRT1 and SIRT3, a nuclear SIRT and a mitochondrial SIRT, respectively, which significantly impact with an increase in mammals’ lifespan by modulating metabolic cellular processes. Particularly, when activated, both SIRT1 and 3 enhance pancreatic β-cells’ insulin release and reduce inflammation and oxidative stress pancreatic damage, maintaining then glucose homeostasis. Therefore, SIRT1 and 3 activators have been proposed to prevent and counteract metabolic age-related diseases, such as type 2 diabetes mellitus (T2DM). Physical activity (PA) has a well-established beneficial effect on phenotypes of aging like β-cell dysfunction and diabetes mellitus. Recent experimental and clinical evidence reports that PA increases the expression levels of both SIRT1 and 3, suggesting that PA may exert its healthy contribute even by activating SIRTs. Therefore, in the present article, we discuss the role of SIRT1, SIRT3, and PA on β-cell function and on diabetes. We also discuss the possible interaction between PA and activation of SIRTs as a possible therapeutic strategy to maintain glucose hemostasis and to prevent T2DM and its complications, especially in the elderly population.

## 1. Introduction: Sirtuins

Sirtuins (SIRTs) belong to a family of nicotinamide adenine dinucleotide (NAD^+^)-dependent protein deacetylases, which act by removing the acetyl groups from several target proteins, such as histones, transcription factors, and cytoplasmic proteins [1]. In humans, seven members of this family exist, SIRT1–7, showing a conserved NAD^+^-binding domain and a catalytic domain and having differences in both N- and C-terminal domains [2].

Sirtuins, besides their multiple functions, regulate several metabolic processes in different tissues [2]. Their cellular functions are also based on their different subcellular localization—SIRT1, SIRT6, and SIRT7 are mainly localized in the nucleus, modulating gene expression [3]; SIRT3, SIRT4, and SIRT5 are localized in mitochondria, where they regulate metabolic and energetic enzymes’ activity [4]. SIRT2 is mainly a cytosolic sirtuin, also shown to even translocate into the nucleus where it modulates cell cycle [5]. Specifically, for the discussed topic of this review, we focused our attention on SIRT1 and SIRT3 among the seven sirtuins, since they are mainly expressed in tissues related to glucose metabolism such as liver, pancreas, heart, skeletal muscles, and adipose tissue, and are the ones exerting a more relevant role in glucose homeostasis [6].

## 2. Sirtuins and Metabolic Diseases: Focus on β-Cell Function

### 2.1. SIRT1

SIRT1 is the most studied sirtuin and regulates several metabolic processes [5]. Among those, an important role of SIRT1 in β-cell function, and in particular in regulating insulin secretion, has been reported [7,8]. Moynihan et al. innovatively reported this evidence by using murine pancreatic β-cells overexpressing SIRT1 (BESTO mice) [7]. Specific SIRT1 overexpression in three-month-old BESTO mice improved both glucose tolerance and insulin release following glucose administration [7]. BESTO mice also exhibited a significant reduction in uncoupling protein 2 (UCP2) expression and a consequent increase in ATP production. Based on previous findings [9], which demonstrated that mice lacking UCP2 showed an improvement in insulin secretion similar to BESTO mice, Moynihan and colleagues concluded that SIRT1-mediated UCP2 repression was responsible for the increased insulin release from β-cells [7]. Moreover, they also reported that improvement in insulin secretion observed in three-month-old BESTO mice could be maintained up to eight months [7]. Decreased β-cell function is one of the leading causes for aging-related diabetes onset [10]; the results obtained by Moynihan et al. suggest that an increase in SIRT1 levels may blunt the age-dependent decline in β-cell function counteracting diabetes in the oldest subjects.

The association between SIRT1 and UCP2 in regulating insulin release was further confirmed and validated by Bordone et al. in the following year [8]. Based on Moynihan et al., the authors reported that SIRT1 knockout mice displayed lower blood insulin levels compared to their control group, confirming the pivotal role of SIRT1 in regulating insulin release [8]. In order to study the mechanisms by which SIRT1 regulates insulin secretion, two different immortalized insulinoma cell lines (rat INS-1 and murine MIN6) were silenced for SIRT1 [8]. Interestingly, in knockdown cells, a decrease in insulin secretion was associated with an enhancement of UCP2 steady-state levels [8]. The most relevant finding reported from this study was that SIRT1 is able to direct bind the UCP2 promoter, repressing its transcription [8]. To further confirm the link between SIRT1 and UCP2, SIRT1 knockdown cells were also silenced for UCP2; the results demonstrated that by repressing UCP2 also in absence of SIRT1, insulin secretion was comparable to control cells [8]. These data highlight a novel insulin secretion pathway that may help to discover novel pharmacological therapies against β-cell dysfunctions.

Consistently with the previous findings on SIRT1 and insulin secretion, Luu et al. reported that SIRT1 also plays a pivotal role in maintaining mitochondrial activity, which leads to a proper insulin secretion release following glucose stimulation [11]. Specifically, both mice SIRT1βKO islets and the murine insulinoma cell line (MIN6) knocked down for SIRT1 showed impaired glucose-stimulated insulin secretion (GSIS) [11]. Moreover, the lack of SIRT1 induced a dysregulation of mitochondrial genes, leading to an alteration in mitochondrial membrane potential, a reduction in mitochondrial oxygen consumption rate, and a decrease in intracellular calcium levels, which explain, at least in part, the compromised GSIS [11]. All these data suggest a relevant implication of SIRT1 in improving GSIS and β-cell function that may result in a potential strategy to help prevent diabetes onset.

Lipotoxicity is considered among the principle causes leading to β-cell dysfunction and promoting insulin resistance and diabetes [12]. A recent study conducted by Desai and colleagues [13] demonstrated that resveratrol, a SIRT1 activator, reverted β-cell dysfunction induced by fatty acid in a rat model [13]. Moreover, to further corroborate the protective role of SIRT1 in β-cells, the authors used the BESTO cell line [13]. Specifically, following induction of fat-mediated β-cell dysfunction, they observed that BESTO cells displayed a reduction in fat-induced damage [13]. These results suggest that SIRT1 may be considered a pharmacological target to counteract the deleterious effect of lipids on β-cell function, which is frequently present in chronic diseases such as obesity and diabetes.

A reduction in β-cell mass, among other factors, contributes to the onset of diabetes [14]. In the last decades, several studies focused on finding novel strategies to improve β-cell mass, and in particular, a recent study reported the involvement of SIRT1 in β-cell regeneration [15]. Specifically, it was shown that SIRT1 activation in mice reduced streptozotocin (STZ)-induced hyperglycemia and attenuated the induced disruption of β-cell mass [15]. Moreover, it was also shown that SIRT1 increased neurogenin3 (NGN3) expression [15], which is responsible for the endocrine cell development in insulin-secreting β-cells [16]. All these findings suggest that SIRT1 may also be considered a novel therapeutic target to improve β-cell regeneration, which is dramatically impaired in diabetic subjects.

### 2.2. SIRT3

The physiopathology of diabetes includes β-cell alterations due to a proinflammatory state and to mitochondrial dysfunction [17,18]. SIRT3 is a mitochondrial protein exerting mainly an anti-inflammatory effect [19]. Caton and colleagues investigated the potential role of SIRT3 activation in protecting β-cell function in diabetic condition [20]. Both SIRT3 mRNA and protein expression was significantly lower in the rat insulinoma cell line (INS-1) after proinflammatory stimuli such as TNFα and IL-1β, markers that are significantly increased in diabetic individuals [17,20]. Accordingly, a relevant decrease in SIRT3 mRNA expression in islets of diabetic subjects compared to their respective control groups was found [20]. Moreover, it has also been demonstrated that SIRT3 knockdown in INS-1 impaired insulin secretion and mRNA levels of several factors involved in insulin synthesis, such as MafA and pancreatic and duodenal homeobox 1 (PDX1) [20], highlighting the relevant role of SIRT3 in maintaining β-cell function. Results from that study also showed that SIRT3 is essential in preserving β-cell mass, since the suppression of SIRT3 significantly increased INS-1 apoptosis [20]. All these data propose SIRT3 as a novel potential therapeutic target to improve β-cell function and mass in diabetic subjects.

As previously reported, lipid accumulation is one of the main risk factors for β-cell dysfunction [12]. In a study conducted by Kim et al., the authors reported the protective role of SIRT3 against palmitate-induced β-cell dysfunction [21]. Particularly, SIRT3 overexpression in murine insulinoma NIT1 cells significantly blunted palmitate-induced apoptosis, as reported by the reduced levels of caspase 3 activity compared to control cells [21]. Interestingly, palmitate reduced ATP production and insulin secretion, whereas SIRT3 enhancement improved both ATP and glucose-stimulated insulin release [21]. Similar results were also found in rat islet overexpressing SIRT3, further validating the important role of SIRT3 in β-cell function [21]. Moreover, since it is well established that palmitate-induced endoplasmic reticulum (ER) stress in β-cells promotes apoptosis and diabetes [22], a significant reduction in mRNA expression of several factors commonly activated by ER stress in overexpressing SIRT3 cells was reported [21]. All these data clearly highlight the protective role of SIRT3 in maintaining β-cell function in lipotoxic conditions, such as diabetes.

## 3. Physical Activity, β-Cell Function, and Diabetes Mellitus

**Figure 1:**
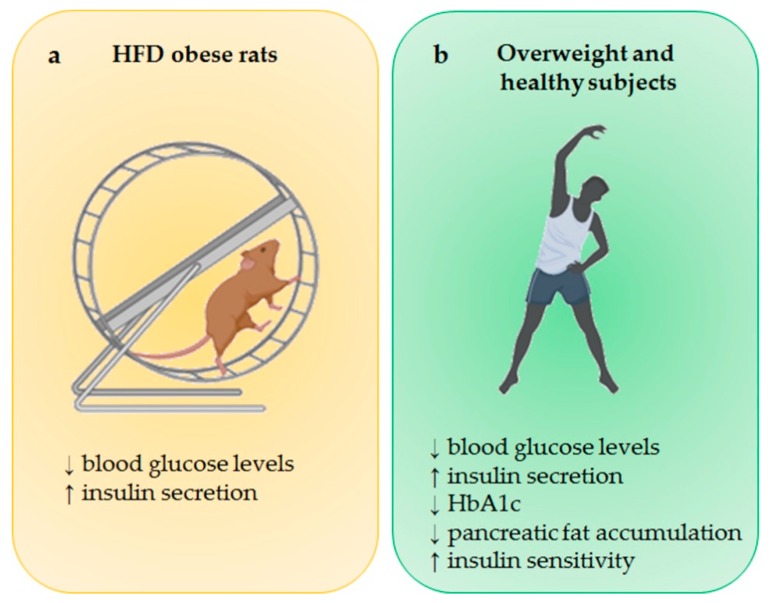
Effect of physical activity (PA) on β-cell function. (**a**) Physical activity reduces blood glucose levels and increases insulin secretion in HFD-induced obese rats compared to sedentary control group. (**b**) In both overweight and healthy subjects, physical activity exerts a beneficial effect on glucose homeostasis, improving insulin secretion and sensitivity and leading to a reduction in HbA1c. BioRender software was used for art work. HFD, high fat diet; HbA1c, hemoglobin A1c.

The beneficial effect of physical activity (PA) on β-cell function and diabetes has been reported in several studies [23,24]. Currently, PA is considered a real first-line therapy against diabetes for its activity in promoting insulin release and protecting against diabetes complications [25]. Diabetes mellitus (DM) is a chronic disease characterized by hyperglycemia due to altered insulin secretion or action, or both [26]. About 90%–95% of DM cases fall into the type 2 DM (T2DM) category, where a peripheral insulin resistance condition is associated with an inadequate insulin release [26]. The other 5%–10% of cases are considered as type 1 DM, where insulin secretion is abolished since β cells are destroyed by an autoimmune process [26]. The long-term complications of DM include cardiovascular disease (CVD), peripheral neuropathy, retinopathy, and nephropathy, among others [26]. Gomes et al. reported that exercise improved β-cell function in proinflammatory high-fat diet (HFD)-induced obese rat [23]. Specifically, animals were divided into four different groups, namely, control sedentary, HFD sedentary, control trained, and HFD trained [23]. Animals were fed with HFD for 10 weeks, with or without 30 sessions of treadmill exercise [23]. Moderate exercise reduced blood glucose levels and significantly improved insulin secretion in HFD-induced obese rats compared to their control sedentary HFD-induced obese rats [23], suggesting that moderate-intensity and low-frequency exercise training positively affects β-cell function and subsequently improves glucose homeostasis (Figure 1a).

Another relevant study conducted by Heiskanen and colleagues reported that exercise training has a beneficial effect on β-cell function and glucose homeostasis in healthy subjects [24]. Particularly, the enrolled individuals were randomly allocated into two different exercise groups for two weeks—the sprint interval training (SIT) consisting of 4–6 episodes of all-out cycling effort for 30 s at maximal workload capacity followed by 4 min recovery, or the moderate-intensity continuous training (MICT) consisting of 40–60 min cycling at 60% of workload capacity [24]. The results showed that both SIT and MICT, by improving muscle glucose uptake and by increasing insulin secretion, significantly reduced the hemoglobin A1c (HbA1c) percentage, exerting a significant beneficial effect on glucose homeostasis [24] (Figure 1b). Moreover, a decrease in pancreas fat content following PA was demonstrated, especially in those subjects showing increased fat accumulation at the enrollment study baseline [24]. Therefore, the study suggests that PA, by improving β-cell function and by reducing pancreas fat content (a leading cause of diabetes), may contribute to decreasing the risk of T2DM.

**Figure 2:**
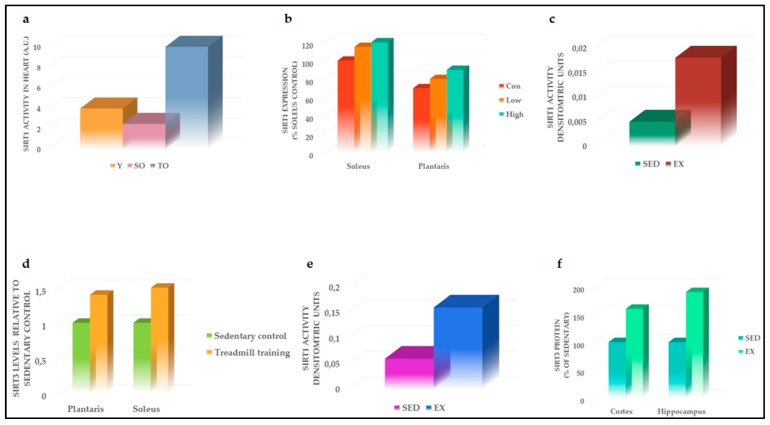
Effects of PA on SIRT1 and SIRT3. (**a**) PA-mediated restoration of SIRT1 activity, in the heart of old rats, modified by Ferrara et al. [32]; (**b**) SIRT1 activity improved in the soleus of rat models following both acute endurance exercise and endurance exercise, modified by Suwa et al. [35]; (**c**) PA increased SIRT1 activity in rats’ ischemic heart, modified by Donniacuo et al. [38]; (**d**) SIRT3 levels increased in soleus and plantaris of rat models compared to sedentary group, modified by Hokari et al. [47]; (**e**) PA increased SIRT3 activity in rats’ ischemic heart, modified by Donniacuo et al. [38]; (**f**) SIRT3 protein expression increased in both cortex and hippocampus of trained mice, modified by Cheng et al. [48]. SIRT1, sirtuin 1; SIRT3, sirtuin 3; Y, young; SO, sedentary old; TO, trained old; Con, control; Low, lower, acute endurance; High, high-endurance training; SED, sedentary; EX, exercise.

Another clinical study conducted in sedentary overweight and dyslipidemic subjects from the Studies of a Targeted Risk Reduction Intervention through Defined Exercise (STRRIDE) randomized control clinical trial demonstrated the positive effect of exercise on β-cell function maintenance [27]. Specifically, all subjects enrolled in this trial were randomly divided into three different exercise training groups, all including treadmill and elliptical trainers for eight months, as follows: (1) high amount/vigorous intensity; (2) low amount/vigorous intensity; (3) low amount/moderate intensity [27]. Although all three types of exercise showed a positive effect on β-cell function, the most significant was related to low amount/moderate-intensity exercise training, which improved β-cell function by inducing a significant increase in insulin sensitivity (Figure 2b), suggesting that this type of exercise training may be the most useful to prevent the onset of diabetes [27].

## 4. Sirtuin Modulation and Physical Activity

PA induces the structural and functional rearrangement of skeletal muscle, and triggers a cluster of body stress responses, in order to maximize the effect of physical exercise [28]. One of the main regulators of this process is NAD^+^, whose levels increase significantly during PA [4]. SIRTs are NAD^+^-dependent histone deacetylases, and when activated they modulate several factors involved in metabolism by controlling both Krebs’ cycle and mitochondrial respiration [28]. Therefore, it is possible to assess that SIRTs may be one of the principle regulators orchestrating tissue changes during PA.

### 4.1. Effects of PA on SIRT1

The effect of PA on SIRT1 levels has been previously investigated by Guerra et al. [29].Specifically, 15 healthy male volunteers were subjected to the Wingate test, a single-bout training consisting of 30 s of all-out exercise on a cycle ergometer [29]. Following the test, a biopsy of the middle portion of the vastus lateralis muscle was performed to evaluate the expression levels of several factors [29]. Among those factors, a significant increase in SIRT1 expression levels 2 h following exercise was reported [29], suggesting an involvement of SIRT1 in modulating the response to acute exercise mainly by an enhancement of mitochondrial oxidative capacity.

Oxidative stress (OS) increases with aging and induced organ dysfunction, such as heart functional changes, among others [30]. Based on the evidence that SIRT1 was a powerful antioxidant able to delay senescence [31], Ferrara et al. conducted a study in rat models to demonstrate that physical exercise, by increasing SIRT1 activity and lowering OS, may counteract the deleterious effect of aging on the heart [32]. Specifically, 24-month-old Wistar rats were acclimated to exercise by walking for 2 weeks. Subsequently, rats were trained with a chronic exercise consisting of running 45 min/day, 5 days/week for 6 weeks [32]. Trained old rats showed a reduction in age-related OS measured using the thiobarbituric acid (TBARS) method [32]. Concurrently, PA restored SIRT1 activity, which resulted in impaired old sedentary rats (Figure 2a) [32], suggesting that the beneficial effect of PA on OS may be mediated by SIRT1 activation. Moreover, the PA-mediated increase in SIRT1 activation induced FOXO3a, which, in turn, is known to regulate the transcription of several factors involved in cell cycle arrest and DNA repair [32]. All these findings highlighted a pivotal antioxidant role of SIRT1 in mediating the beneficial effects of chronic PA.

The involvement of SIRT1 in regulating the mitochondrial oxidative capacity, following PA, has been also evaluated in human skeletal muscle [32]. Based on Ferrara and colleagues [32], Gurd et al. reported an increase in SIRT1 activity after six weeks of high-interval training (HIT) in the skeletal muscle of nine healthy volunteer subjects, in association with enhanced protein expression levels of SIRT1 target proliferator-activated receptor gamma coactivator 1-alpha (PGC-1α) [33]. Since PGC-1α regulates mitochondrial biogenesis [34], these data suggest that SIRT1 activation during exercise promoted mitochondrial biogenesis in order to increase the oxidative capacity of muscle in response to PA.

Based on the role of SIRT1 in muscle adaptation during PA, Suwa and colleagues evaluated the effects of acute endurance exercise and low- or high-intensity exercise training on SIRT1 and its intracellular targets, such as PGC-1α [35]. Old male rats were acclimated to PA for three days and then subjected to lower, acute endurance or high-endurance training. Levels of both SIRT1 and PGC-1α were enhanced in slow-twitch oxidative muscle (soleus), where they promoted mitochondrial biogenesis maintaining muscle oxidative ability [35]. Furthermore, while SIRT1 expression was enhanced during endurance exercise in the fast-twitch muscle (plantaris), no significant modulation was observed for PGC-1α [35], suggesting that the muscle adaptive effect of PA-related was mainly due to SIRT1 activity rather than PGC-1α. However, the authors suggested that different types of endurance exercise might induce PGC-1α expression, leading to muscle adaptation [35]. SIRT1 activity significantly increased in the soleus after both acute endurance exercise and endurance exercise (Figure 2b) [35]. Since it is well established that active SIRT1 regulates several factors involved in mitochondrial respiration and fatty acid oxidation [36], SIRT1 activation may contribute to muscle adaptation to PA.

Huang and collaborators reported a positive association between PA and SIRT1 in different aged rat groups [37]. Specifically, they examined the effects of 40 min/day of swimming exercise training for 12 weeks in young (3 months old), middle-aged (12 months old), and old rats (18 months old). A significant increase in SIRT1 levels in both gastrocnemius and soleus mainly in mice aged 3 months and 12 months was evident, compared to control sedentary groups [37]. A relevant enhancement in trained animals was also reported for PGC-1α, confirming that different endurance exercise training may modulate PGC-1α expression, which is directly regulated by SIRT1, according to a previously discussed study [35], confirming the pivotal role of SIRT1- PGC-1α axes in regulating muscle adaptation to PA. The lack of increase in SIRT1 after PA in old animals, even if 18-month-old animals are still considered middle-aged, may suggest lower SIRT1 levels and activity during aging, that if reestablished might revert the beneficial effect of SIRT1 on muscle tissue. Studies including older rats (24 months old) and SIRT1 activators combined with PA are needed to confirm this hypothesis.

The protective effect of exercise mediated by SIRT1 has also been investigated in the heart of infarcted rats [38]. CVDs are among the most important complications and the first cause of deaths in diabetes [39]. Rats trained with running at 30 m/min, 45 min/day, 5 days/week, for 6 weeks showed a significant reduction in myocardial damage following myocardial infarction (MI) compared to the sedentary group. Moderate prolonged PA increased SIRT1 nuclear localization leading to a decrease in apoptotic marker expression, such as Bax and caspase 3, induced by MI [38]. These findings are of particular importance since they describe for the first time the molecular pathways involved in the cardioprotective effect of SIRT1-dependent PA, further confirming the relevant beneficial non-pharmacological role of PA in activating SIRT1 (Figure 2c).

Diabetes and alteration in glucose homeostasis are significantly linked with higher risk for cognitive impairment and overt dementia, since Alzheimer’s disease is also known as type 3 diabetes [40]. It is also clearly established that PA exerts a protective effect in the brain by improving cognitive function, such as memory and learning, throughout the induction of neurogenesis and the enhancement in cerebral blood flow [41,42]. These PA effects are mediated by an increase in brain-derived neurotrophic factor (BDNF) expression in the hippocampus [43]. Although several factors have been described to regulate BDNF [44,45], El Hayek and colleagues aimed to identify novel exercise-mediated molecules able to ameliorate cognitive function via BDNF [46]. Mice were subjected to voluntary running for 30 days and then lactate and BDNF were evaluated. As expected, levels of both analyzed factors increased in the trained group compared to the control mice [46]. Moreover, lactate injection in exercised mice improved spatial memory formation as assessed by performing the Morris water maze test compared to exercised mice injected with lactate transporter inhibitor [46]. Interesting, BDNF induction was dependent on SIRT1 activation since the inhibition of SIRT1 resulted in decreased BDNF levels and reduced neuronal plasticity [46]. Therefore, PA-induced SIRT1 activation may also be a good target to prevent neurodegenerative disorders in diabetic patients.

### 4.2. Effects of PA on SIRT3

Mitochondria are the leading energy suppliers of the body, in particular in muscle cells [49]. The main sirtuin that presents in mitochondria, which in part regulates their function, is SIRT3 [50]. In a study conducted by Hokari and colleagues, the effects of endurance exercise training on SIRT3 levels in rat skeletal muscle were tested [47]. Male Wistar rats were acclimated to running for 1 week and then were trained with 20 m/min for 60 min, 7 days/week for 3 weeks [47]. This protocol of training increased SIRT3 levels in soleus and plantaris (Figure 2d), whereas the voluntary exercise enhanced SIRT3 expression in triceps and plantaris but not soleus. This result may be explained by the fact that forelimb muscles are mainly recruited and activated during voluntary running rather than during treadmill exercise [51]. Moreover, SIRT3 expression was modulated by muscle contractile activity; in fact, when mice hindlimbs were immobilized, SIRT3 steady-state levels were significantly reduced [47].

As reported for SIRT1 [38], Donniacuo and colleagues demonstrated a cardioprotective effect of SIRT3 in trained infarcted rats [38]. Particularly, PA preceding MI significantly increased SIRT3 levels (Figure 2e) and SIRT3-dependent antioxidants, such as catalase and manganese superoxide dismutase (MnSOD) [38]. The increase in SIRT3-mediated anti-oxidative pathways, in turn, significantly blunted the damages induced by MI, whereas its effect was not present in the sedentary group [38]. SIRT3 is therefore able to induce protection against MI induced by oxidative damage following coronary artery occlusion. Thus, PA, acting through SIRT3 activation, may be considered a potential non-pharmacological antioxidant therapy against cardiovascular diseases in diabetic patients.

In a study conducted in healthy young and old volunteer subjects, it was reported that endurance training (ET) increased SIRT3 levels [52]. Specifically, the ET group was subjected to 8 weeks of cycling for 60 min, 3 to 5 times a week [52]. In the control groups, SIRT3 levels decreased in old compared to young subjects [52]. However, following exercise, SIRT3 levels in muscles significantly increased in both young and old subjects [52]. Moreover, it was demonstrated that SIRT3 enhancement deacetylated isocitrate dehydrogenase 2 (IDH2) mitochondrial protein, whose acetylation increases mitochondrial oxidative stress [53]. These findings highlight the pivotal role of PA-induced SIRT3 levels in counteracting the increase in mitochondrial oxidative stress observed in the elderly.

Another study conducted in 19 obese adolescent males demonstrated that PA increased SIRT3 levels [54]. Specifically, all participants were subjected to moderate-intensity aerobic training for 12 weeks without changes in caloric intake [54]. The results showed that SIRT3 levels increased following PA; moreover, a positive association between SIRT3 and PGC1α was reported [54], suggesting that PA enhanced mitochondrial function in a SIRT3-dependent manner.

In the previous section, we discussed the neuroprotective effect of PA mediated by SIRT1 activation. Similarly, SIRT3 counteracted the excitotoxicity induced by OS observed during PA [48]. Particularly, wild-type mice subjected to exercise (wheel running) for one month showed a significant increase in protein and mRNA levels of SIRT3 in both hippocampus and cerebral cortex, compared to their sedentary control groups (Figure 2f) [48]. SIRT3 enhancement induced the deacetylation of superoxide dismutase 2 (SOD2), suggesting the activation of this antioxidant enzyme as the leading mechanism in the reduction of reactive oxygen species (ROS) levels generated during PA. Moreover, the SIRT3-mediated deacetylation and subsequent inactivation of cyclophilin D was also reported, promoting ATP production and restoring mitochondrial membrane potential [48]. All these findings suggested that exercise-mediated SIRT3 activation is essential in order to counteract the adverse effect of OS produced by the increased mitochondrial activity during PA, thereby sustaining body health.

According to the evidence on the neuroprotective effect of SIRT3 during exercise, Shi and colleagues demonstrated that aerobic interval training (AIT)-induced SIRT3 expression decreased OS generated by HFD, thereby improving cognitive function [55]. The authors reported that HFD significantly compromised cognitive function in mice and particularly in SIRT3-deficient mice [55], remarking that alterations in glucose and lipid metabolisms are noxious for vital organs. Moreover, HFD increased acetylation levels of several proteins in the hippocampus; among those, MnSOD became iper-acetylated and was unable to blunt ROS production [55]. Subsequently, mice fed with HFD were subjected to AIT for 6 weeks, showing an increase in SIRT3, which, in turn, deacetylated MnSOD and reduced OS [55]. Furthermore, AIT improved spatial cognition and memory ability in HFD-induced obese mice [55]. AIT-mediated SIRT3 activation exerted a protective role in hippocampal neurons, preventing cognitive decline in obese individuals and opening novel thoughts in this field.

## 5. Tandem Effect of Sirtuin 1 and 3 Activation and PA in Maintaining Glucose Homeostasis and Counteracting Diabetes Complications

A correlation between PA, SIRT1 activation, and glucose metabolism has already been reported by Suwa et al. [35]. Specifically, the authors showed that low- and high-intensity endurance exercise training increased SIRT1 and glucose transporter 4 (GLUT4) expression levels in both soleus and plantaris of rat models [35]. These data suggest that endurance exercise, by inducing GLUT4 protein expression, may improve insulin sensitivity in a SIRT1-dependent manner.

The experimental and clinical evidence previously discussed suggests that PA may be considered as a real therapy in terms of cure and prevention of several chronic diseases [56]. However, as a real therapy, PA must be prescribed with attention and using a right approach, especially in patients with chronic diseases. An inadequate type and/or duration of PA may have several risks per se, including an increase in OS (due to the higher consumption of oxygen during exercise), in glucose consumption (leading to hypoglycemia with important complications in diabetic patients), risk for falls in the elderly population, risk for bone, muscle, and joint damages in older people, alteration in heart rhythms, among others [57]. Therefore, having a deep understanding of how healthy effects can be achieved through PA, and of the pathways underlying PA’s physiological role in our body system, is pivotal to employing it at its best and to obtaining the maximum level of benefit.

SIRT1 and 3 have been strongly implicated in regulating aging processes and associated with age-related diseases, especially by regulating metabolism [6]. Diabetes, in particular T2D, and its complications, increase dramatically with age [58]. The alteration in β-cell function along with the increase in insulin resistance leading to impairment in glucose homeostasis in old patients are among the most important mechanisms correlated with the higher incidence of diabetes in that population [58]. A decrease in SIRT1 and 3 expression and activity has been reported in aged animals and in older humans as well [6]. In the present review, we reported that PA increases the levels of these SIRTs and therefore may be helpful in counteract aging and senescent phenotypes, by modulating either these enzymes or their salvage correlated pathways. However, as we know, elderly subjects may have limitations in performing the entirety of the PA necessary to increase SIRT levels, due to phenotypes of frailty and other associated age-related limitations, such as sarcopenia (loss in skeletal muscle mass and function) [59].

**Figure 3:**
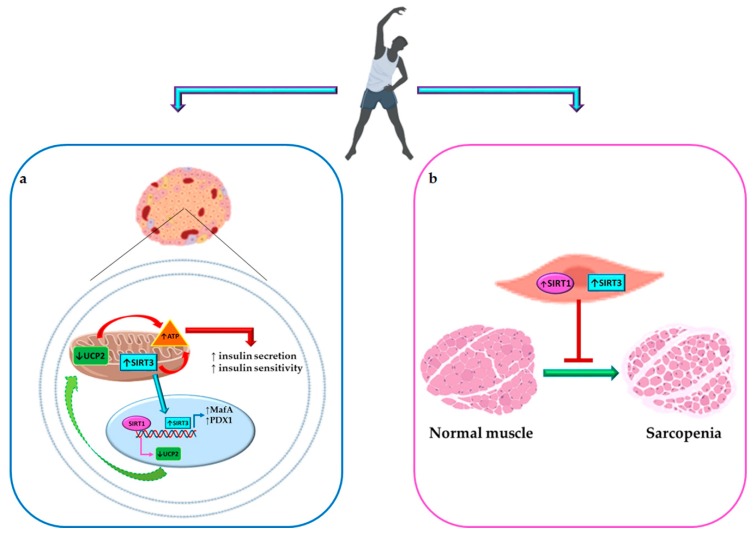
SIRT1- and SIRT3-mediated effects of physical activity (PA) on metabolism. (**a**) PA exerts a beneficial effect on glucose metabolism by inducing SIRT1 and SIRT3. Specifically, SIRT1 reduces UCP2 levels leading to an increase in ATP production, enhancing both insulin secretion and insulin sensitivity. Moreover, SIRT3 induces ATP release and the transcription of MafA and PDX1, regulating both insulin secretion and insulin synthesis respectively. (**b**) PA, by inducing SIRT1 and SIRT3 expression, significantly blunted the progression of sarcopenia in skeletal muscle related to both aging and diabetes. BioRender software was used for art work. SIRT1, sirtuin 1; UCP2, uncoupling protein 2; ATP, adenosine triphosphate; SIRT3, sirtuin 3; PDX1, pancreatic and duodenal homeobox 1.

For instance, an increase in sarcopenia is strictly associated with sedentary behavior and is a disease induced by muscle insulin resistance and impairment in glucose homeostasis [60]. Recently, Myers and colleagues demonstrated, by using SIRT1 knockout mice, that SIRT1 presence is pivotal to avoiding muscle fatigue and sarcopenia [61]. Similar findings have been reported for SIRT3 for its role in mitochondria [62]. In human aging, sedentariness is associated with SIRT3 expression reduction in skeletal muscle with consequent sarcopenia in the elderly population, but PA enhances SIRT3 expression and eliminates differences between young and older individuals [63]. Therefore, activation of SIRT1 and 3 may avoid aging-associated sarcopenia and further help individuals to exercise, which in turn may further increase SIRT1 and 3, provoking a beneficial loop that may counteract aging and its related diseases (Figure 3).

## 6. Conclusions

In conclusion, the main question is how may we activate SIRTs and prevent metabolic dysfunction, especially in the aging population? Recently, the Copenhagen Consensus statement 2019 on PA and aging defined physical activity and exercise as follows: PA entails body movement that increases energy expenditure relative to rest, and is often characterized in terms of intensity from light, to moderate to vigorous; and Exercise as a subset of structured PAs that are more specifically designed to improve cardiorespiratory fitness, cognitive function, flexibility balance, strength, and/or power [59]. Therefore, as we mentioned, it would be difficult to restore metabolic impairment associated with an age-related decrease of SIRTs using only PA modulation. Functional activators of SIRTs have been proposed and the major ones are natural products, such as resveratrol, which are contained in common foods [64]. The intake of several food groups, such as red meat and sugar-sweetened beverages, is positively associated with T2DM and impairment in glucose homeostasis [65]. Based on that fact and on the evidence discussed in this review suggesting a combination of well-designed and controlled PA and a healthy diet, such as the Mediterranean diet that is rich in polyphenols [66], we may conclude that this is the best approach to having a beneficial impact on SIRT levels in order to control and prevent metabolic disorders.

## Abbreviations

AIT	Aerobic interval training
ATP	Adenosine triphosphate
BDNF	Brain-derived neurotrophic factor
CVD	Cardiovascular disease
DM	Diabetes mellitus
ER	Endoplasmic reticulum
ET	Exercise training
GLUT4	Glucose transporter 4
GSIS	Glucose-stimulated insulin secretion
HbA1c	Hemoglobin A1c
HFD	High-fat diet
IDH2	Isocitrate dehydrogenase 2
IL-1β	Interleukin 1 beta
MI	Myocardial infarction
MICT	Moderate-intensity continuous training
MnSOD	Manganese superoxide dismutase
NAD^+^	Nicotinamide adenine dinucleotide
NGN3	Neurogenin3
OS	Oxidative stress
PA	Physical activity
PDX1	Pancreatic and duodenal homeobox 1
PGC-1α	Peroxisome proliferator-activated receptor gamma coactivator 1 alpha
ROS	Reactive oxygen species
SIRT	Sirtuin
SIT	Sprint interval training
STZ	Streptozotocin
T2DM	Type 2 diabetes mellitus
TBARS	Thiobarbituric acid
TNFα	Tumor necrosis factor alpha
UCP2	Uncoupling protein 2
